# Serum Positive for the Autoantibody against the ***β***_1_-Adrenoceptor from Chinese Patients with Congestive Heart Failure Decreases *I*_ss_ in Mouse Cardiac Myocytes

**DOI:** 10.1155/2011/143517

**Published:** 2011-06-07

**Authors:** Yuan-yuan Wang, Zhi-Yong Ma, Xiao-Dong Li, Jian-chun Wang, Wei Zhang, Li Li, Yun Zhang

**Affiliations:** ^1^Key Laboratory of Cardiovascular Remodeling and Function Research, Chinese Ministry of Education and Chinese Ministry of Public Health, Jinan 250012, China; ^2^Department of Cardiology, QiLu Hospital, Shandong University, Jinan 250012, China; ^3^Department of Cardiology, Shandong Provincial Hospital, Shandong University, Jinan 250021, China

## Abstract

Autoantibodies targeting the *β*_1_-adrenergic receptor (AAB-*β*_1_) display agonist-like effects, which may have a pathogenic role in the progression of heart failure. Here, we used the electrophysiological recordings to explore the effects of AAB-*β*_1_-positive serum from Chinese patients with heart failure on the activity of the peak transient outward potassium current (*I*_to_) and the end 50 ms steady-state potassium current (*I*_ss_) in mouse cardiac myocytes. We found that the AAB-*β*_1_-positive serum had no effect on the activity of *I*_to_, but it produced a decrease in the currents of *I*_ss_. A low concentration of positive serum (1/100) had a small inhibitory effect on *I*_ss_. However, positive serum at 1 : 10, 1 : 20, and 1 : 50 significantly decreased *I*_ss_. The concentration-dependence analysis showed that the EC_50_ of AAB-*β*_1_-positive serum was 1/60.24 and its nH was 2.86. It indicated that the AAB-*β*_1_ could inhibit *I*_ss_ in mouse cardiomyocyte in a concentration-dependent manner.

## 1. Introduction

It has become increasingly clear that autoimmune disorders are a feature of congestive heart failure (CHF) of various etiologies [1, 2]. Over the past few decades, several autoantibodies have been detected in the serum of patients with CHF, including autoantibodies against *α*-adrenoceptor [3, 4], *β*_1_-adrenoceptor [5–9], and M_2_-adrenoceptor [10–12]. Autoantibodies targeting the second extracellular loop of the *β*_1_-adrenergic receptor (AAB-*β*_1_) are specifically associated with the effects of *β*-blocker therapy and correct prediction of ventricular tachycardia and sudden death in patients with idiopathic dilated cardiomyopathy [8, 13]. *In vivo*, AAB-*β*_1 _can induce *β*_1_-adrenergic receptor uncoupling, which causes cardiomyocyte apoptosis and sustained calcium influx that results in cardiac electrical instability [14]. These results suggest that AAB-*β*_1_ displays agonist-like effects that may have a pathogenic role in the progression of heart failure. 

Additional evidence has revealed the electrophysiological effects of AAB-*β*_1_. AAB-*β*_1_ and the IgG fraction containing this antibody significantly enhanced *I*_ca_ amplitude of adult rat ventricular myocytes [15]. The previous study also showed that purified autoantibodies enhanced cell shortening, prolonged action potential duration, and increased calcium current amplitude of rat ventricular myocytes; these positive effects of AAB-*β*_1_ were indeed mediated via the *β*_1_-adrenoceptor [14, 16]. However, the effects of AAB-*β*_1_ on voltage-gated potassium channels in mouse ventricular myocytes remained unclear.

In cardiac myocytes, voltage-gated K^+^  currents are responsible for the repolarization of the membrane potential and, therefore, influence action potential duration (APD). Previous studies have described a steady state outward K^+^  current aside from the transient outward K^+^  current in rat ventricular cells [17–19]. This steady state current displayed a weak voltage-dependent inactivation and was negatively regulated by the *β*-adrenergic agonist isoprenaline. Thus, this steady state current might play an important role in determining APD during neurohormonal regulation. Therefore, we explored the effects of AAB-*β*_1_-positive serum from Chinese patients with CHF on the activity of the peak transient outward potassium current (*I*_to_) and the end 50 ms steady state potassium current (*I*_ss_) in mouse cardiac myocytes.

## 2. Materials and Methods

### 2.1. Patients

Fourteen patients admitted to the Department of Cardiology of QiLu Hospital of Shandong University with stable CHF enrolled and submitted to serological tests, coronary angiography, and electro- and echocardiography to discard those with the following pathological conditions: Chagas' disease, hypertrophic cardiomyopathy, acute coronary syndrome, severe hypertension, valvular heart disease, alcohol or drug abuse, insulin-dependent diabetes mellitus, and severe infection. All selected patients had left ventricular ejection fractions (LVEF) ≤45% determined by echocardiography (*M* mode). They were receiving standard therapy, including angiotensin-converting enzyme inhibitors or angiotensin receptor blockers, diuretics, and digitalis glycosides during the study. None of them were being treated with *β*-blockers at enrollment. Five control sera were obtained from voluntary healthy blood donors. The blood was collected and fractioned, and the serum was stored at −20°C until immunological and/or electrophysiological assays were performed. This study was performed in compliance with the Declaration of Helsinki, and the protocol was approved by the ethics committee of QiLu Hospital. All patients gave informed consent for participation. 

### 2.2. Autoantibodies

The target peptide was a fusion protein corresponding to the putative sequence of the second extracellular loop of the human *β*_1_-adrenergic receptor (amino acids 197 to 222: H-W-W-R-A-E-S-D-E-A-R-R-C-Y-N-D-P-K-C-C-D-F-V-T-N-R), which was commercially synthesized. Peptide purity was ascertained by mass spectroscopy analysis. The presence of autoantibodies was determined by ELISA. ELISA was carried out as previously described [12, 20] with the following modifications: the wells of microtiter plates were coated with this peptide (10 *μ*g/mL) and incubated for 2 hours. After washing the plate 3 times, 100 *μ*L of 3% skim milk was added to each well for 2 hours. Then 100 *μ*L of patient serum (at dilutions starting from 1 : 20) was added to the coated wells of the microtiter plate. After washing the plate 3 times, an affinity-purified antihuman immunoglobulin G peroxidase-conjugated antibody (diluted 1 : 5000) was added to each well for 1 hour. After washing the plates 4 more times, bound peroxidase-conjugated antibody were detected by incubation with the chromogenic substrate for peroxidase. The reaction was stopped with 50 *μ*L of sulfuric acid, and the optical density was determined at 450 nm. A positive reaction was defined as ≥2.5 times the background level. Autoantibodies directed against the *β*_1_-adrenergic receptor were detected in 6 patients (43%) by ELISA.

### 2.3. Cell Isolation

Ventricular myocytes were dissociated from the hearts of mice according to previously published protocol [14]. Briefly, 8-week-old male Kunming mice (30–40 g) were anaesthetized with pentobarbitone sodium (30–40 mg/kg), which were injected intravenously together with heparin (100 IU/kg). The heart was removed, washed in a cold calcium-free Joklik MEM (Sigma) solution, and perfused for 5 min on a Langendorff apparatus with the same calcium-free Joklik MEM (containing 11.0 g/L Joklik MEM and 10 mmol/L HEPES, the pH was adjusted to 7.3 with NaOH) warmed to 37°C. The heart was then perfused with collagenase-containing solution (collagenase*Ⅱ*,1 mg/mL, Worthington, and BSA 1 mg/mL). After approximately 15 min, the ventricles were removed, placed in fresh solution, cut into 1 mm^3^ sections, and gently agitated to dissociate the myocytes. Single ventricular myocytes were collected in KB solution (composition in mM: 30 KCl, 35 KOH, 3 MgSO_4_, 50 L-glutamic acid, 0.5 EGTA, 20 taurine, 10 glucose, and 10 HEPES; pH adjusted to 7.2 with KOH). Cells were stored at 22–24°C.

### 2.4. Electrophysiological Recordings

The cardiac myocytes were transferred to a recording chamber mounted on an inverted microscope (NIKON TE2000-U) at least 10 min before patch clamping. Micropipettes were made from borosilicate glass capillary with an outside diameter of 1.5 mm. After being fire-polished and filled with pipette solution (composition in mM: 115 K-aspartate, 5 KCl, 4 Na_2_ATP, 7 MgCl_2_, 5 EGTA, and 10 HEPES; pH was adjusted with NaOH to 7.2), the resistance was 2–4 MΩ. The junction potential between the patch pipettes and bath solution was nullified immediately before GΩ seal formation. Cell capacitances were read from the potentiometer to set transient capacitances to zero. After the pipette and cell transient capacitance were compensated, the membrane was ruptured with gentle suction to obtain the whole cell voltage-clamp configuration using PCS-5200 micro-operation (Burleigh, USA). Signals were amplified with HEKA EPC-10 patch clamp amplifier and controlled with the Pulse software (HEKA, Lambrecht, Germany). Signals were sampled at 3 kHz and filtered at 1 kHz. The voltage protocol was a 1-s depolarizing step from −50 to +50 mV in 10 mV increments from a holding potential of −60 mV. The peak of the current was the transient outward potassium channel current (*I*_to_), and the end 50 ms of plateau potential current was *I*_ss_. All experiments were performed at room temperature (22–25°C). The ventricular myocytes were perfused with normal bath solution (BS, composition in mM: 135 ChCl, 5.4 KCI, 1.2 MgCl_2_, 0.5 CdCl, 10 glucose, and 10 HEPES; pH was adjusted to 7.4 with NaOH) for 10 min to stabilize the currents. For analysis of autoantibody effects, cells were separately perfused with BS including AAB-*β*_1_-negative serum and AAB-*β*_1_-positive serum for 5 min. For the concentration-dependence analysis of autoantibody effects, cells were perfused with the following bath solutions: serum dilution ranging from 1/100, 1/50, 1/20 to 1/10. 

The recordings were analyzed using IGOR and the Origin software. The value of current was expressed with the density of current (pA/pF) to eliminate the capacitance error. Current amplitude was determined as the difference between peak inward current and current at the end of the depolarising step.

### 2.5. Statistics

All of the data were presented as the means ± S.E. One-way ANOVA with repeated measures and analysis of variance were used for statistical analysis where appropriate. Statistical analysis was performed using the SPSS12.0 software, and *P* < .05 was considered statistically significant. AAB-*β*_1_-positive serum dilution-response curves were fitted using the equation: *I* = *a*/(1 + (EC_50_/dilution)^*n*H^  ), where *a* was the amplitude of the *I*_ss_ current, the EC_50_ was the dilution where a half-maximal response was induced, and *n*H was the Hill coefficient.

## 3. Results

**Figure 1:**
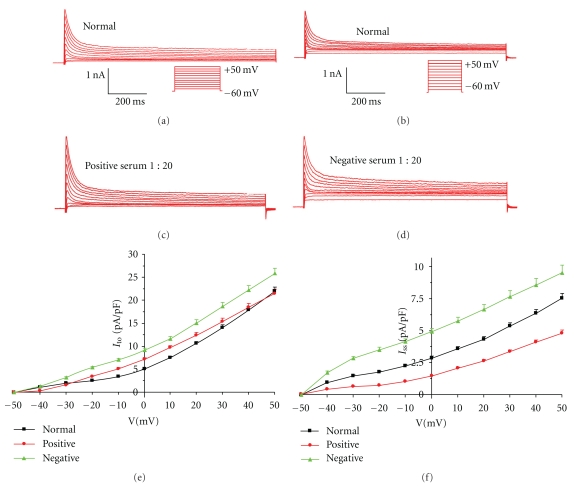
Effect of AAB-*β*_1_-negative and -positive serum of CHF patients on outward K^+^  currents of mouse cardiomyocytes: (a), (b), (c), and (d), current traces obtained with 1-s depolarizing step from −50 to +50 mV in 10 mV increments from a holding potential of −60 mV. Voltage protocols are shown below the current traces. Under the present experimental conditions, the peak of the current was *I*_to_, and the end 50 ms of plateau potential current was *I*_ss_. (e), (f), corresponding current–voltage relationships during the application of AAB- *β*_1_-negative or -positive serum of CHF patients at the dilution of 1 : 20. AAB-*β*_1_ negative serum had no effect on the activity of *I*_to_, *I*_ss_ and the corresponding current-voltage curves (b, d, e, f). The AAB- *β*_1_-positive serum had no effect on activity of *I*_to_, but it produced a decrease in the currents of *I*_ss_ (a, c, e, f).

Under these experimental conditions (in the presence of ChCl and CdCl to block the Na^+^ currents and Ca^2+^ currents, resp.), outward K^+^  currents were recorded in mouse myocytes. These readings were composed of rapidly activating and inactivating currents (*I*_to_) and slowly activating but noninactivating current (*I*_ss_) (Figure 1(a), 1(b), 1(c), 1(d)).

**Figure 2:**
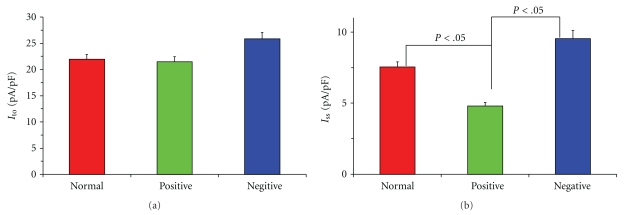
Comparison of AAB-*β*_1_-negative serum and -positive serum from patients with heart failure (dilution at 1 : 20) on the mean current of outward K^+^  currents at a voltage of 50 mV. Compared to currents at normal bath solution and AAB-*β*_1_-negative serum (*n* = 5 cells, dilution at 1 : 20), AAB-*β*_1_-positive serum (*n* = 7 cells, dilution at 1 : 20) had no effect on *I*_to_  (*P* > .05). *I*_ss_ on the AAB-*β*_1_-positive serum decreased significantly compared to the normal bath solution (*P* < .05) and had also significant decrease compared to the AAB-*β*_1_-negative serum (*P* < .05).

The AAB-*β*_1_-negative serum of CHF patients had no effect on the activity of *I*_to_ and *I*_ss_ in mouse ventricular myocytes (Figures 1(b) and 1(d)). The negative serum also showed no effect on the current-voltage curves of *I*_to_ and *I*_ss_ (Figures 1(e) and 1(f)). The AAB-*β*_1_-positive serum had no effect on activity of *I*_to_, but it produced a decrease in the currents of *I*_ss_ (Figure 1(a), 1(c), 1(e), 1(f)). Compared to the currents at normal bath solution and AAB-*β*_1_-negative serum (dilution at 1 : 20), AAB-*β*_1_-positive serum (dilution at 1 : 20) had no effect on *I*_to_ but caused a significant decrease in *I*_ss_ myocyte currents (*P* < .05, Figure 2).

**Figure 3:**
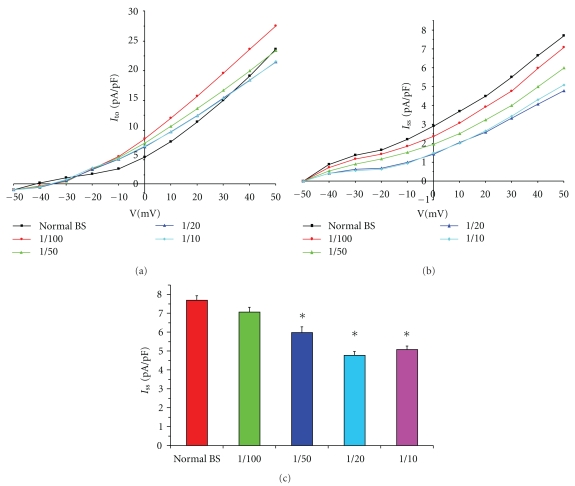
Different concentrations of AAB-*β*_1_-positive serum (dilution ranging from 1 : 100, 1 : 50, 1 : 20 to 1 : 10) on the outward K^+^  currents at a voltage of 50 mV: *I*_to_ showed no changes at different concentrations of AAB-*β*_1_-positive serum (a). A low concentration of AAB-*β*_1_-positive serum (1 : 100) had a small inhibitory effect on *I*_ss_ (b, c). However, AAB-*β*_1_-positive serum at 1 : 10, 1 : 20, and 1 : 50 significantly decreased *I*_ss_ (*P* < .05, b, c). Additionally, there were no significant differences in currents at 1/10 and 1/20 AAB-*β*_1_-positive serum treatment (b, c). AAB-*β*_1_-positive serum had no effect on the *I*-*V* relationship at any concentration (a, b). *P < .05.

**Figure 4:**
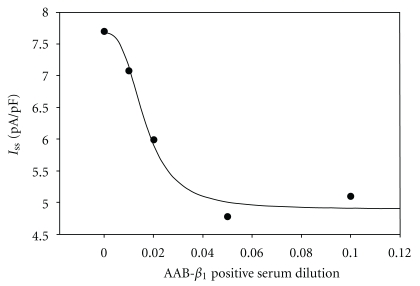
Concentration-dependent inhibition of AAB-*β*_1_-positive serum on *I*_ss_ activated at a potential of 50 mV: EC_50_ (dilution of AAB-*β*_1_ positive serum that produces the half-maximum blockade) of AAB-*β*_1_-positive serum was 1 : 60.24 and nH was 2.86.

Compared with the normal bath solution, the current density of *I*_to_ showed no change at different concentrations of AAB-*β*_1_-positive serum (Figure 3(a)). A low concentration of AAB-*β*_1_-positive serum (1/100) had a small inhibitory effect on *I*_ss_ (Figures 3(b) and 3(c)). However, AAB-*β*_1_-positive serum at 1 : 10, 1 : 20 and 1 : 50 significantly decreased *I*_ss_ (*P* < .05, Figures 3(b) and 3(c)). Additionally, there were no significant differences in the *I*_ss_ currents between 1/10 and 1/20 AAB-*β*_1_-positive serum (Figures 3(b) and 3(c)) treatments. Similarly, AAB-*β*_1_-positive serum had no effect on the *I*-*V* relationship at any concentration (Figures 3(a) and 3(b)). The concentration-dependence analysis showed that the EC_50_ of AAB-*β*_1_-positive serum was 1/60.24, and its nH was 2.86 (Figure 4). 

## 4. Discussion

Increasing evidence demonstrates that the contribution of AAB-*β*_1_ to the pathogenesis of chronic heart failure is not just a correlation. In the present study, we found for the first time that serum positive for autoantibodies against the *β*_1_-adrenoceptor decreases the current density of *I*_ss_  in mouse ventricular myocytes in a concentration-dependent manner, with no effect on *I*_to_. AAB-*β*_1_-positive serum at the dilution of 1 : 10, 1 : 20, and 1 : 50 significantly decreased *I*_ss_. Concentration-dependence analysis showed that the EC_50_ was 1/60.24 and *n*H was 2.86.

The autoantibodies for the *β*_1_-adrenergic receptor have been found in sera not only from patients with idiopathic dilated cardiomyopathy [5], but also from patients with CHF of various etiologies [21, 22]. Previous studies have conclusively demonstrated that autoantibodies targeting the second extracellular loop of the *β*_1_-adrenergic receptor showed agonist-like effects: inducing receptor uncoupling, causing cardiomyocyte apoptosis, and permitting sustained calcium influx [14, 23]. In the present study, serum positive for autoantibodies against the *β*_1_-adrenoceptor decreased the current density of *I*_ss_ without any effect on *I*_to_, which is similar to the inhibitory effect of the *β*_1_-adrenergic agonist isoprenaline [17]. From this close resemblance of macroscopic *I*_ss_ after stimulation with AAB-*β*_1_ and isoprenaline, we suggest that both activators mediate their effects via similar signal transduction pathways. 

Autoantibodies are thought to induce activation of the receptor that leads to intracellular signaling involving the classical PKA pathway [24–27]. Other groups have reported effects of purified autoantibodies or AAB-*β*_1_-positive serum on calcium channels. Christ et al. found that immunoglobulin G derived from patients positive for the *β*_1_-adrenoceptor autoantibodies increased Ca^2+^ current to a similar extent, but prolonged the plateau of duration of action potentials to a lesser extent compared to isoprenaline [14]. However, Del Corsso et al. found that serum from patients with IDC induced a significant decrease in isoproterenol-stimulated L-type Ca^2+^ currents in rabbit ventricular myocytes. This activation is known to involve the PKA pathway [12]. Furthermore, Christ et al. concluded that AAB-*β*_1_ may not only enhance *I*_Ca_ via stimulation of the *β*_1_-adrenoceptors, but may also inhibit this *β*_1_-adrenoceptor-mediated increase upon stimulation with catecholamines [16]. In our study, AAB-*β*_1_-positive serum inhibited *I*_ss_ in a concentration-dependent manner with no effect on the current-voltage curves. Therefore, the regulatory effect of AAB-*β*_1 _on ion channel currents may all involve the classical PKA pathway in the different studies.

Furthermore, AAB-*β*_1_-positive serum only decreased *I*_ss_ with no effect on *I*_to_, which was similar to the results of *β*_1_-adrenergic agonist isoprenaline treatment [17]. Several hypotheses can be proposed to account for such a difference in threshold dose and potency. The channels may be more easily accessible to phosphorylation in *I*_ss_ than *I*_to_, which is a possible effect according to the theory of cAMP compartmentalization. Another possibility is that the channels may be more sensitive to phosphorylation in *I*_ss_ than *I*_to_; for example, phosphorylation at one site on *I*_ss_ may be sufficient to induce an effect while *I*_to_ requires phosphorylation of several sites. The above suggested mechanisms may also lead to the dose-dependence of *I*_ss_.

## 5. Conclusions

Autoantibodies against *β*_1_-adrenoceptor from Chinese patients with congestive heart failure can inhibit *I*_ss_ in mouse cardiomyocytes in a concentration-dependent manner. Because *I*_ss_ plays an important role in the repolarization of action potentials, AAB-*β*_1_ may influence action potential duration via this current.

### 5.1. Study Limitations and Clinical Implications

In the present study, we did not investigate the mechanism behind the inhibitory effect of AAB-*β*_1 _on *I*_ss_. It would require much more work to establish whether AAB-*β*_1_ inhibits *I*_ss_ directly or indirectly. Recent studies reported that AAB-*β*_1_ may influence the effects of *β*-blocker therapy and that specific removal of AAB-*β*_1_ by immunoadsorption can improve cardiac function in patients with DCM. These results suggest that anti-*β*_1_-adrenergic receptor autoantibodies have a pathogenic role in the onset and progression of heart failure. Because *I*_ss_ has biophysical properties of being slowly activated and noninactivated (steady state), AAB-*β*_1_ may prolong repolarization and action potential duration by inhibiting *I*_ss_; this would subsequently result in cardiac electrical instability.
